# Nintedanib allows retreatment with atezolizumab of combined non-small cell lung cancer/idiopathic pulmonary fibrosis after atezolizumab-induced pneumonitis: a case report

**DOI:** 10.1186/s12890-019-0920-9

**Published:** 2019-08-22

**Authors:** Hideaki Yamakawa, Tomohiro Oba, Hiroki Ohta, Yuta Tsukahara, Gen Kida, Emiri Tsumiyama, Tomotaka Nishizawa, Rie Kawabe, Shintaro Sato, Keiichi Akasaka, Masako Amano, Kazuyoshi Kuwano, Hidekazu Matsushima

**Affiliations:** 1 Department of Respiratory Medicine, Saitama Red Cross Hospital, 1-5 Shintoshin, Chuo-ku, Saitama, Saitama 330-8553 Japan; 2 Department of Respiratory Medicine, Tokyo Jikei University Hospital, Tokyo, Japan

**Keywords:** Nintedanib, Immune checkpoint inhibitors, Drug-induced pneumonitis

## Abstract

**Background:**

Nintedanib is a tyrosine kinase inhibitor that efficiently slows the progression of idiopathic pulmonary fibrosis (IPF) and has an acceptable tolerability profile. In contrast, immune checkpoint inhibitors (ICIs) such as programmed death 1 and programmed death ligand 1 inhibitors have shown clinical activity and marked efficacy in the treatment of non-small cell lung cancer. However, it is unclear whether nintedanib reduces the risk of ICI-induced pneumonitis in IPF.

**Case presentation:**

A 78-year-old man with squamous cell lung carcinoma in IPF underwent second-line treatment with pembrolizumab. He was diagnosed as having pembrolizumab-induced pneumonitis after two cycles. He was administered prednisolone (PSL) and then improved immediately. Thereafter, his lung cancer lesion enlarged despite treatment with TS-1. Atezolizumab was then administered as 4th-line chemotherapy, but he immediately developed atezolizumab-induced pneumonitis after 1 cycle. The re-escalated dosage of PSL improved the pneumonitis, and then nintedanib was started as additional therapy. Under careful observation with nintedanib, atezolizumab was re-administered on day 1 of an every-21-day cycle. After three cycles, it remained stable without exacerbation of drug-induced pneumonitis.

**Conclusion:**

This case indicates the possibility that the addition of nintedanib to ICI therapy might prevent drug-induced pneumonitis or acute exacerbation of IPF. However, whether anti-fibrotic agents such as nintedanib are actually effective in preventing ICI-induced pneumonitis in ILD remains unknown and additional research is greatly needed to identify effective therapies for ILD combined with lung cancer.

## Background

The treatment of advanced non-small cell lung cancer (NSCLC) has evolved to include targeted therapy, immune checkpoint inhibitors (ICIs), and chemotherapy for selected patients in the first-line setting. Angiogenesis inhibitors have been used in combination with chemotherapy in the first-line and maintenance settings to provide improved progression-free survival, objective response rate, and overall survival in selected studies. A biologic rationale exists for combining anti-angiogenic agents with immunotherapy and targeted kinase inhibitors [1]. ICIs aid in enhancing antitumor activities, and as a byproduct they can also stimulate the immune system, resulting in immune-related adverse events such as ICI-related pneumonitis. This is contributed to by patients’ smoking history, damage to underlying lung parenchyma, chronic obstructive pulmonary disease, and pulmonary fibrosis [2–5].

Nintedanib is a tyrosine kinase inhibitor that efficiently slows the progression of idiopathic pulmonary fibrosis (IPF) and has an acceptable tolerability profile [6]. Treatment with nintedanib reduces the risk of acute exacerbations (AEs), and a combined analysis of data from clinical trials of nintedanib shows a trend towards a reduction in mortality [7]. Moreover, a study such as J-SONIC is ongoing to evaluate the efficacy and safety, including AE of IPF (AE-IPF), of nintedanib combined with cytotoxic drugs compared with cytotoxic drugs alone for chemotherapy-naïve patients with IPF combined with NSCLC [8]. However, it is unclear whether nintedanib reduces the risk of ICI-induced pneumonitis of IPF. We herein report a case of NSCLC combined with IPF in which recurrence of ICI-induced pneumonitis may have been prevented with nintedanib therapy.

## Case presentation

### Case report

**Fig. 1:**
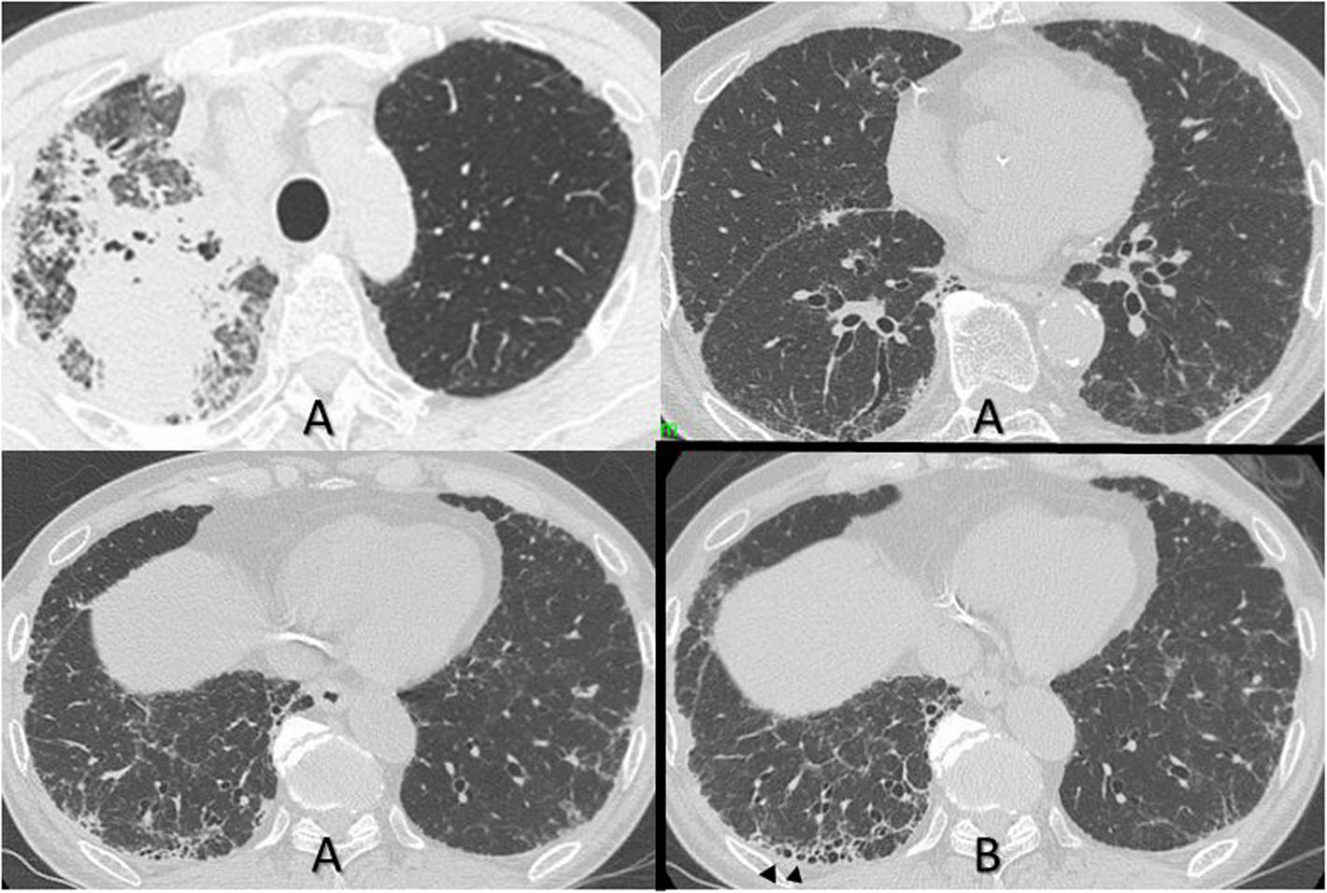
(**a**) Chest high-resolution computed tomography performed at initial presentation showed a mass lesion in the right upper lobe as the primary lung cancer and interstitial abnormality predominantly in the lower lobe. The interstitial abnormality was basal predomoinant and showed reticulation with peripheral traction bronchiectasis and bronchioloectasis, which was probably compatible with usual interstitial pneumonia pattern. (**b**) One year and 3 months after initial presenteation, honeycomb lesions appeared in the lower lobe (arrowheads)

We present the case of a 78-year-old man, a former smoker, with squamous cell lung carcinoma. Clinical staging was stage IV [cT3N2M1c (ADR)]. He was simultaneously diagnosed as having interstitial pneumonia. Chest high-resolution computed tomography (CT) showed a mass lesion of the right upper lobe as the primary lung carcinoma that was surrounded by ground-glass opacities as carcinomatous lymphangiomatosis. Interstitial pneumonia, as indicated by a subpleural reticular shadow with traction bronchiectasis and bronchiolectasis predominantly in the lower lobes and without apparent honeycombing, was comparable with probable usual interstitial pneumonia pattern based on recent criteria [9] (Fig. 1a). He had no symptoms suspicious of connective tissue disease and serological domain as all auto-antibodies. In addition, he had no history of exposure-evoked aspects of chronic hypersensitivity pneumonitis or familial or chronic drug-induced pneumonitis.

**Fig. 2:**
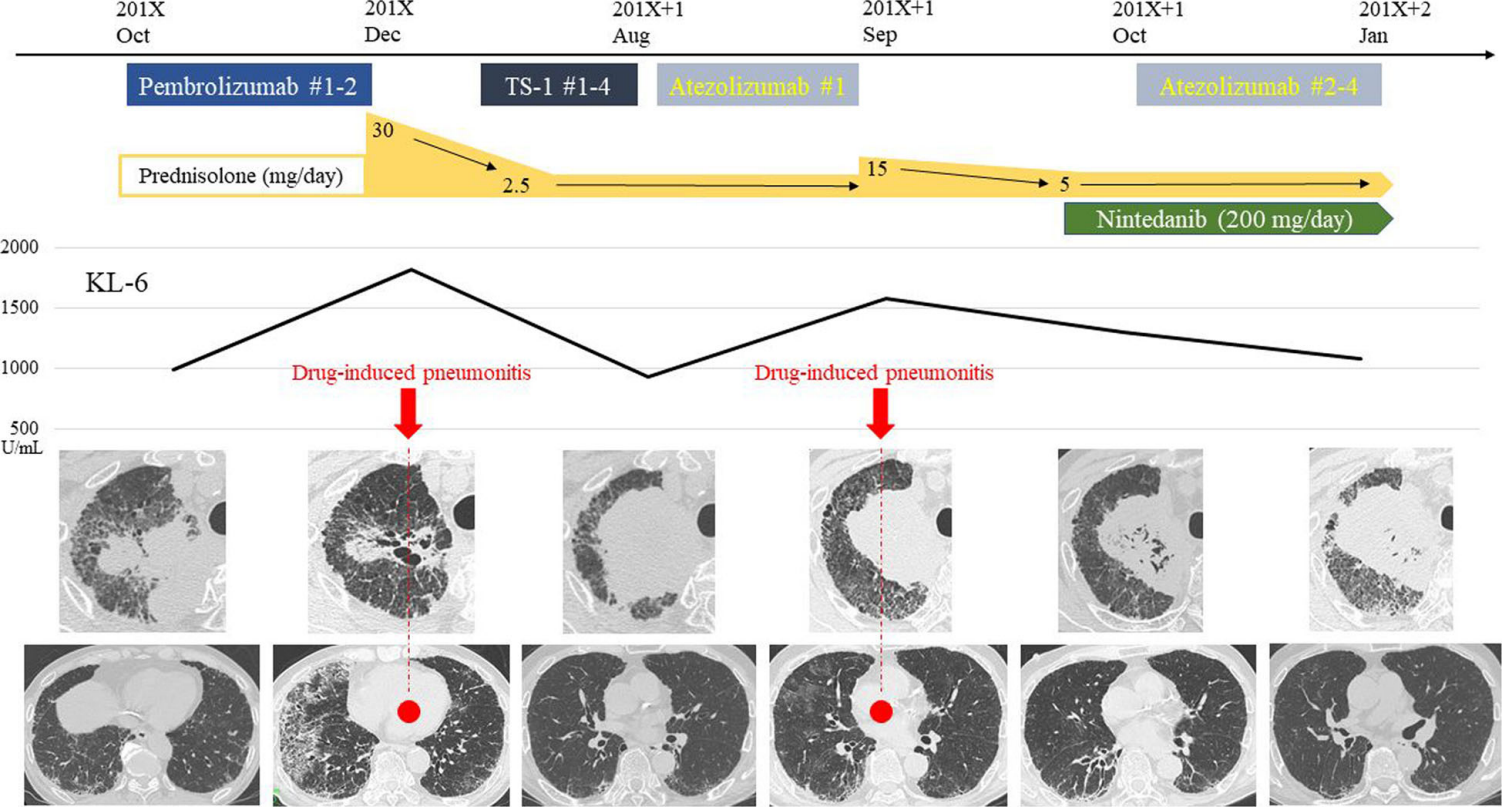
Changes over time in Krebs von den Lungen-6 (KL-6) and the pulmonary lesion on chest computed tomography (CT). The patient underwent second-line treatment with pembrolizumab. He was diagnosed as having pembrolizumab-induced pneumonitis after two cycles because CT revealed bilateral ground-glass opacities and his serum levels of KL-6 were increased, although the size of the lung cancer tumors had decreased. He was administered prednisolone (PSL), after which his KL-6 levels decreased and the pneumonitis immediately improved. However, his lung cancer lesion continued to enlarge despite treatment with TS-1. Atezolizumab was then administered as fourth-line chemotherapy, but he developed atezolizumab-induced pneumonitis after 1 cycle. The re-escalated dosage of PSL improved his pneumonitis, and the serum levels of KL-6 decreased, and then nintedanib was started as additional therapy. Under careful observation with nintedanib (200 mg/day) and PSL (5 mg/day), atezolizumab was re-administered on day 1 of an every-21-day cycle. After three cycles, it remained stable without exacerbation of drug-induced pneumonitis

The patient underwent first-line treatment with carboplatin and nab-paclitaxel from May 201X. After 4 cycles, disease progression was recognized. Therefore, second-line chemotherapy of pembrolizumab was administered. However, CT revealed bilateral ground-glass opacities and his serum levels of Krebs von den Lungen-6 (KL-6) were increased, although the size of the lung cancer tumors was reduced after 2 cycles (Fig. 2). We diagnosed pembrolizumab-induced pneumonitis and then started prednisolone (PSL) at 30 mg/day. This pulmonary toxic lesion began to improve immediately, and the dose of PSL was gradually tapered to 2.5 mg/day in April 201X + 1. Because of apparent regrowth of the cancerous lesion with brain metastasis, stereotaxic radiation of brain lesions and tegafur/gimeracil/oteracil (TS-1) as third-line chemotherapy were administered. However, disease progression was confirmed. At one year and 3 months after his initial presentation (August 201X + 1), honeycomb lesions appeared in the lower left lobe (Fig. 1b). Therefore, we diagnosed IPF because the radiological disease course became one of typical usual interstitial pneumonia and the patient had no evidence indicating other etiologies.

After careful examination of the drug-induced pneumonitis, atezolizumab was administered as fourth-line chemotherapy, but the patient developed subacute atezolizumab-induced pneumonitis after 1 cycle. A re-escalated dosage of PSL improved his pneumonitis, and his serum levels of KL-6 decreased, following which nintedanib was started as additional therapy for the IPF. Under careful observation with nintedanib (200 mg/day) and PSL (5 mg/day), atezolizumab was re-administered on day 1 of an every-21-day cycle. After three cycles, it remained stable without exacerbation of drug-induced pneumonitis. However, afterwards, he developed multiple brain metastases and carcinomatous meningitis. Because his therapy was converted to palliative care, he died one and a half months later due to carcinomatous meningitis.

## Discussion and conclusion

Our patient developed drug-induced pneumonitis from both pembrolizumab and atezolizumab. However, the use of nintedanib allowed us to re-administer atezolizumab without further recurrence of the drug-induced pneumonitis. We highlight the possibility of using nintedanib to prevent atezolizumab-induced pneumonitis in IPF combined with lung cancer. ICIs such as programmed death 1 (PD-1) and PD ligand 1 inhibitors have shown clinical activity and marked efficacy in the treatment of NSCLC [10]. Pembrolizumab as a PD-1 inhibitor and atezolizumab as a PD ligand 1 inhibitor were used in our patient. Certain ICI-related adverse events have been observed related to the skin, gastrointestinal tract, endocrine system, liver, lungs, and kidneys [11, 12]. Drug-induced pneumonitis has been reported as one of the most common lung-related immunological adverse events with an incidence ranging between 1 and 5% [10]. With nivolumab as a PD-1 inhibitor, the clinical course of most patients with nivolumab-induced pneumonitis was relatively good following the cessation of nivolumab and the initiation of a corticosteroid. It is noteworthy that two thirds of patients with nivolumab-induced pneumonitis were able to restart their nivolumab therapy [13]. However, one third of patients developed recurrent pneumonitis. It is not clear which subgroup among the patients with ICI-related pneumonitis will suffer the recurrence of pneumonitis. However, existing interstitial pneumonia is a known risk factor of ICI-related pneumonitis [2–5]. Moreover, AE-IPF, as in our case, is a severe and life-threatening complication [14]. AE-IPF is triggered by various causes such as infection, post-procedural/postoperative period, drug toxicity, and aspiration [14]. Although the direct relevance remains unknown, ICIs may be an important trigger in AE-IPF. Our patient developed drug-induced pneumonitis with both pembrolizumab and atezolizumab, and moreover, the atezolizumab-induced pneumonitis occurred immediately. Because of this clinical course and co-existing IPF, we thought our patient was at high risk for recurrence of serious ICI-induced pneumonitis or AE-IPF. Therefore, we hypothesized that nintedanib might allow re-administration of atezolizumab without recurrence of drug-induced pneumonitis.

Nintedanib has a number of clinical benefits in patients with IPF, such as reducing the decline of lung function and extending the time to AE [7]. Nintedanib is an oral angiokinase inhibitor that targets receptors in three proangiogenic pathways: vascular endothelial growth factor (VEGF) receptors, platelet-derived growth factor receptors α/β, and fibroblast growth factor receptors. Therefore, this drug has been expected to have an anti-tumor effect on lung cancer [15]. In fact, combination therapy with nintedanib and docetaxel significantly improved independently assessed progression-free survival compared with placebo/docetaxel in the overall LUME-Lung-1 study population and provided significant, clinically meaningful improvement in overall survival in patients with adenocarcinoma [16]. This study showed that the rates of occurrence of drug-induced pneumonitis did not differ between the nintedanib and placebo groups (1.4 and 0.8%, respectively). However, the J-SONIC trial, a randomized control study for the treatment of NSCLC associated with IPF, showed that nintedanib combined with carboplatin plus nab-paclitaxel prolonged the interval to AE-IPF compared with carboplatin plus nab-paclitaxel alone [8]. That is, nintedanib was thought to be a key drug in the treatment of lung cancer combined with IPF. On the other hand, the excessive autoimmune response of tumor infiltrating lymphocytes can be the one of the reasons of ICI-induced pneumonitis [17]. VEGF can act as an immunosuppressive factor by several mechanisms such as inhibiting dendric cell (DC) function and maturation, enhancing expression of PD-L1 by DCs, promoting into the tumor [18]. Therefore, nintedanib that the target the VEGF pathway may enhance the prevention of ICI-induced pneumonitis. Unfortunately, the combination of nintedanib with atezolizumab showed no anti-tumor effectiveness in our case. However, although there have never been trials of combination treatment of ICIs and nintedanib until now, to the best of our knowledge, this combination therapy may improve the safety and survival of lung cancer associated with IPF.

In conclusion, our case indicates the possibility that ICIs combined with nintedanib might prevent drug-induced pneumonitis or AE-IPF. However, whether anti-fibrotic agents such as nintedanib are actually effective in the prevention of ICI-induced pneumonitis in ILD remains unknown, and additional research is greatly needed to identify effective therapies for ILD combined with lung cancer.

## Data Availability

The data will not be shared with participant confidentiality.

## Abbreviations

AE: Acute exacerbation
CT: Computed tomography
DC: Dendric cell
ICIs: Immune checkpoint inhibitors
IPF: Idiopathic pulmonary fibrosis
KL-6: Krebs von den Lungen-6
NSCLC: Non-small cell lung cancer
PD-1: Programmed death 1
PD-L1: Programmed death ligand 1
PSL: Prednisolone
VEGF: Vascular endothelial growth factor
